# ¹H, ¹³C, and ¹⁵N backbone resonance assignments of zinc-α2-glycoprotein, a lipid-mobilizing protein structurally related to class I HLA molecules

**DOI:** 10.1007/s12104-026-10283-4

**Published:** 2026-11-09

**Authors:** Elizabeth A. Corbin, Uri Z. Miles, Hongwei Wu, Andrew C. McShan

**Affiliations:** School of Chemistry and Biochemistry, Georgia Institute of Technology, Atlanta, 30332 USA

**Keywords:** NMR resonance assignment · ZAG · major histocompatibility complex

## Abstract

Zinc-α2-glycoprotein (ZAG) is a soluble, secreted protein that shares a fold with class I major histocompatibility complex (MHC-I) molecules but does not present antigens to T cells. Instead, ZAG leverages its MHC-I-like groove to bind a diverse set of metal ions, lipids, proteins, and nucleic acids to function in lipid mobilization, metabolism, and cellular signaling. The structural basis of ZAG’s ligand interactions in solution remains poorly understood. Here, we report near-complete (94.6%) backbone (¹H^N^, ¹⁵N, ¹³Cα, ¹³Cβ, ¹³C′) resonance assignments of perdeuterated, uniformly [²H, ¹⁵N, ¹³C]-labeled human ZAG (32 kDa non-glycosylated form), refolded *in vitro* from *E. coli* inclusion bodies and assigned using 3D TROSY-based solution-state NMR experiments. The chemical shifts have been deposited in the Biological Magnetic Resonance Data Bank (BMRB ID: 54025). Backbone amide-amide NOEs and secondary structure predicted by TALOS-N agree closely with the previously published ZAG crystal structure. Comparison with the classical MHC-I molecule HLA-A*02:01 reveals that ZAG’s helical domains and β-sheet platform are similarly rigid and well-ordered in solution, even though ZAG lacks a bound peptide and β2-microglobulin and has diverged functionally. The reported resonance assignments provide a foundation for future NMR studies of ZAG’s structure, biomolecular interactions, conformational dynamics, and biochemical function.

**Supplementary Information:**

The online version contains supplementary material available at https://doi.org/10.1007/s12104-026-10283-4.

## Biological Context

Zinc-α2-glycoprotein (ZAG, gene name *AZGP1*) is a member of the non-classical class I major histocompatibility complex (MHC-I) protein family (Maenaka and Jones 1999). ZAG was first isolated from human plasma following the addition of zinc acetate and was subsequently characterized through genetic and biochemical studies (Burgi and Schmid 1961; Ohkubo et al. 1988; Araki et al. 1988; Sánchez et al. 1997a). The ZAG amino acid sequence is evolutionarily conserved across practically all mammals (Freije et al. 1993). ZAG contains 278 residues after removal of its signal peptide and has a molecular weight of approximately 32 kDa and 42 kDa in its non-glycosylated and glycosylated forms, respectively (Kamboh and Ferrell 1986; Ohkubo et al. 1990). ZAG is expressed in a variety of locations, including the liver, prostate, breast, stomach, testes, kidney, and adipose tissue (Tada et al. 1991; Bao et al. 2005). Unlike classical MHC-I molecules, which are displayed at the cell surface by their transmembrane domain, ZAG is a soluble protein secreted into biological fluids, such as seminal fluid, saliva, sweat, urine, milk, plasma, and prostatic fluid (Poortmans and Schmid 1968; Bundred et al. 1987). The atomic structure of ZAG has been determined by X-ray crystallography, revealing a striking resemblance to classical MHC-I molecules (Bjorkman et al. 1987; Sánchez et al. 1999). Similar to MHC-I, ZAG features a ligand-binding groove formed by α1 and α2 helices atop a platform of pleated β-sheets, alongside an α3 domain that folds into an immunoglobulin-like β-sandwich. However, unlike MHC-I molecules, which require β_2_-microglobulin (β_2_M) for proper folding and stability, ZAG does not associate with β_2_M and folds independently (Sánchez et al. 1997b). Despite its homology to classical MHC-I, ZAG does not appear to bind peptide antigens nor activate T cells.

Rather than acting as an antigen-presenting molecule, ZAG serves as a multifunctional protein with roles in lipid mobilization, lipid and carbohydrate metabolism, fertility, cancer, inflammation, epilepsy, and obesity (Hassan et al. 2008b; McDermott et al. 2012; Wei et al. 2019; Banaszak et al. 2021). ZAG-knockout mice are predisposed to weight gain, and reduced ZAG expression in obese individuals has been linked to insulin resistance and diabetes (Rolli et al. 2007; Gong et al. 2009; Yang et al. 2013; Zhang et al. 2020). These effects have largely been attributed to ZAG’s lipolytic activity in adipocytes via β3-adrenergic receptor-mediated regulation of hormone-sensitive lipases and other lipid-metabolizing enzymes (Hirai et al. 1998; Gong et al. 2009; Bing et al. 2010; Russell and Tisdale 2012; Zhang et al. 2020). In cancer, alterations in ZAG expression have been observed in breast, liver, prostate, gastric, colorectal, and lung malignancies, where ZAG can either promote or suppress tumor progression depending on the cellular context (Díez-Itza et al. 1993; Hale et al. 2001; Falvella et al. 2008; Yip et al. 2011). For example, ZAG overexpression in tumor can drive cancer cachexia through excess lipolysis and downstream lipotoxicity (Bing et al. 2004; Mracek et al. 2011). Additionally, ZAG has been implicated in suppressing epileptic seizure activity, potentially through modulation of neuroinflammation, ketogenesis, and blood-brain barrier integrity via GLUT-3, mTOR, MAPK, and TGF-β signaling pathways (Liu et al. 2018; Peng et al. 2021).

The remarkable functional diversity of ZAG stems from its ability to interact with a wide range of partners, including metal ions, lipids, proteins, and nucleic acids, via its MHC-I-like binding groove. *In vitro* binding studies have shown that the ZAG binding groove accommodates synthetic lipid-like molecules, such as PEG, DAUDA, and C_16_-BODIPY, as well as natural long-chain polyunsaturated fatty acids, such as docosahexaenoic acid, eicosapentaenoic acid, and arachidonic acid (Kennedy et al. 2001; Delker et al. 2004; Zahid et al. 2022). Polyunsaturated fatty acids and other lipid molecules are likely endogenous ligands linked to ZAG’s lipid mobilization abilities. Crystal structures of ZAG bound to PEG and DAUDA have been reported (Delker et al. 2004; Lau et al. 2019), but information regarding its endogenous lipid-binding modes and lipid specificity remain largely lacking. ZAG has also been reported to directly interact with a surprising variety of protein partners, including β3-adrenergic receptor (Russell and Tisdale 2012), prolactin-inducible protein (Hassan et al. 2008a), ERK2 / TGF-β1 (Liu et al. 2017), filamin A (Ji et al. 2019), TRIM25 (Yun et al. 2024), acylglycerol kinase (Qiu et al. 2024), amine oxidase copper-containing 3 (Romauch 2020), and others. While a crystal structure of ZAG bound to prolactin-inducible protein is available (Hassan et al. 2008a), biophysical and structural insights into how ZAG engages its protein partners are also lacking. In addition, ZAG has been identified as an RNA-binding protein that associates with circular RNA and long non-coding RNA overexpressed in cancer (Gong et al. 2023; Yuan et al. 2023). Relatedly, ZAG possesses intrinsic ribonuclease activity, suggesting a broader capacity to regulate transcriptome stability (Lei et al. 1998). The molecular basis for ZAG’s RNA-binding and nuclease activity, like that of its many protein and lipid interactions, remains structurally and biophysically undefined. Finally, zinc ions play important, albeit mysterious, roles in ZAG’s cellular functions (Kumar et al. 2013; Banaszak et al. 2021). ZAG seems to be able to bind to other metal ions in the Irving-Williams Series (Ullah et al. 2020), although how metal ions contribute to ZAG’s structure and function remains an open question.

Detailed solution-state characterization of ZAG would greatly facilitate mapping its metal ion, lipid-, protein-, and nucleic acid-binding surfaces, while revealing the conformational dynamics and binding specificity underlying its multifunctionality. However, NMR resonance assignments for ZAG have not been previously reported. Here, we report the backbone resonance assignments of human ZAG (32 kDa non-glycosylated form), prepared as a perdeuterated protein refolded *in vitro* after expression in *Escherichia coli*. We compare chemical shift-derived secondary structural index and random coil index order parameter values of ZAG with the class I MHC-I molecule HLA-A*02:01. The work provides a foundation for future solution-state NMR-based investigations of ZAG’s structure, biomolecular interactions, conformational dynamics, and biochemical functions.

## Methods and experiments

### Expression and purification of perdeuterated ZAG

Recombinant plasmid DNA encoding for full-length human ZAG lacking its N-terminal signal peptide (residues 21–298; Uniprot ID #P25311) was subcloned into pET-22b(+) with NdeI and XhoI restriction sites. Plasmid DNA (~ 100 ng) was transformed into *E. coli* BL21(DE3) competent cells by heat shock and plated onto LB-agar containing 50 µg/mL carbenicillin. U-[^2^H, ^15^N, ^13^C]-labeled ZAG was expressed in 1 L of D_2_O-based 1× M9 minimal media [final concentrations: 47.8 mM Na_2_HPO_4_, 22 mM KH_2_PO_4_, 8.6 mM NaCl, 1 g/L ^15^NH_4_Cl (Cambridge Isotope Laboratories), 3 g/L D-Glucose-^13^C-1,2,3,4,5,6,6-D₇ (Cambridge Isotope Laboratories), 1 mM MgSO_4_, 0.1 mM CaCl_2_, 1× MEM vitamins, 1× Trace Metals, 25 µg/mL carbenicillin, 0.2 g/L ISOGRO-^13^C,^15^N,^2^H (Sigma-Aldrich)] (Azatian et al. 2019). Transformed colonies were grown in 60 mL of D_2_O-based 1× M9 minimal media starter culture for 16 h at 37 °C at 225 RPM. The starter culture was added to 1 L of D_2_O-based 1× M9 minimal media and the culture was grown at 37 °C at 225 RPM until an OD_600_ of 0.6. ZAG expression was induced with 1 mM isopropyl-β-D-thiogalactopyranoside and the culture was allowed to grow at 37 °C at 225 RPM for an additional five hours. Cells were harvested at 6,300 RPM for 20 min at 4 °C. The bacterial cell pellet was resuspended in 12.5 mL BugBuster (Millipore) containing 100 mM phenylmethylsulfonyl fluoride. The cell suspension was sonicated at 40% amplitude on ice in 30 s cycles for a total of 6 min followed by centrifugation for 20 min at 10,000 RPM at 4 °C. Supernatant was discarded and the inclusion body pellet was isolated by sonication in 25 mL of Wash Buffer A [100 mM Tris pH 8, 2 mM EDTA, 0.1% w/v deoxycholate] followed by 25 mL of TE Buffer [100 mM Tris pH 8, 2 mM EDTA] with centrifugation steps for 20 min at 10,000 RPM at 4 °C in between washes. Inclusion bodies were resuspended in 6 mL TE buffer with sonication. For inclusion body solubilization, 5.7 g guanidine hydrochloride and 360 µL of fresh 0.1 M dithiothreitol were added. The solution was incubated for one hour at room temperature with gentle agitation then centrifuged at 10,000 RPM for 20 min at 4 °C to remove insoluble material. Solubilized inclusion body concentration was measured with a NanoDrop spectrophotometer at 280 nm. The molecular weight and molar extinction coefficients used for calculations were obtained from ExPASy ProtParam (Wilkins et al. 1999).

*In vitro* refolding was performed as previously described (Kumar et al. 2013). Briefly, 50 mg of ZAG inclusion bodies were added in increments of 500 µL over two days to 250 mL of refolding buffer [100 mM Tris pH 8, 2 mM EDTA, 0.4 M Arginine-HCl, 4.9 mM L-glutathione reduced, 0.57 mM L-glutathione oxidized] while stirring at 4 °C. The refolding mixture was left at 4 °C without stirring for two days. No ligands were added to the refolding buffer, resulting in “apo” ZAG. Refolded ZAG was dialyzed into 5 L of dialysis buffer [150 mM NaCl, 25 mM Tris pH 8] using dialysis membrane with a 3500 Da molecular weight cutoff (Spectrum) for 18 h at 4 °C with stirring. Refolded ZAG was concentrated and purified by size-exclusion chromatography using an ÄKTA Go FPLC with a HiLoad 16/600 Superdex 75 pg column with a flow rate of 1 mL/min into SEC buffer [20 mM sodium phosphate pH 6.5, 100 mM NaCl]. Protein purity was verified by SDS-PAGE.

### Biophysical analysis of *in vitro* refolded ZAG

Far-UV circular dichroism (CD) was collected on a JASCO J-815 spectropolarimeter. CD was measured from 190 nm to 350 nm with 0.5 mg/mL ZAG in CD buffer [20 mM NaCl, 20 mM sodium phosphate pH 6.5] using a 1 mm path length quartz cuvette (Starna Cells #21-Q-1/CD). CD was acquired in triplicate at 25 °C with a scan rate of 50 nm/min and then averaged. The average CD spectra were smoothed using a Savitzky-Golay filter with convolution width of 9. CD signal in mdeg was converted into molar ellipticity using the equation [θ] = mdeg × mres / 10 × d × c.

Nano differential scanning fluorometry (nanoDSF) was performed using a Prometheus NT.48 (NanoTemper Technologies). ZAG at 10 µM in nanoDSF buffer [100 mM NaCl, 20 mM sodium phosphate pH 6.5] was loaded into nanoDSF grade standard capillaries (NanoTemper Technologies) and exposed to temperatures ranging from 20 °C to 95 °C with a thermal ramping rate of 1 °C/min. The ratio of intrinsic fluorescence emission (F_350_ nm/F_330_ nm) was monitored. Melting temperature (T_m_) were obtained from fitting the first derivative plots in the Prometheus NT.48 Software. Three replicates were acquired and the data is presented as the mean ± standard deviation (*n* = 3).

### Solution NMR data acquisition and processing

A sample of 600 µM U-[^2^H, ^15^N, ^13^C]-labeled *in vitro* refolded human ZAG in NMR buffer [100 mM NaCl, 20 mM sodium phosphate pH 6.5, 1 mM EDTA, 0.02% w/v NaN_3_, 10% v/v D_2_O, 1× cOmplete protease inhibitor (Roche)] was used for backbone assignments. Backbone resonances (^1^H_N_, ^15^N, ^13^Cα, ^13^Cβ, and ^13^C’) of ZAG were assigned using TROSY-based triple-resonance experiments acquired with non-uniform sampling (NUS) of approximately 20% Poisson-gap sampling. 3D NMR data acquired with NUS was reconstructed with istHMS (Hyberts et al. 2012, 2014). The following experiments were recorded: 2D amide TROSY-HSQC (Bruker pulse sequence trosyf3gpphsi19), 3D TROSY-HNCA (Bruker pulse sequence trhncagp2h3d2), 3D HNCO (Bruker pulse sequence trhncogp2h3d), 3D TROSY-HNCACB (Bruker pulse sequence trhncacbgp2h3d), and 3D TROSY-HN(CO)CACB (Bruker pulse sequence trhncocacbgp2h3d) (Salzmann et al. 1998; Eletsky et al. 2001). Acquisition times were 79.8 msec in ^1^H, 20.5 msec in ^15^N, 22.7 msec in ^13^CO, 20.5 msec in ^13^C_α_, and 5.2 msec in ^13^C_β_. Recycle delay was set to 1.5 s in all 3D experiments. All solution NMR data were acquired at a ^1^H field of 800 MHz at 25 °C on a Bruker AVIIIHD-800 spectrometer equipped with a 3 mm TCI cryoprobe. To obtain through-space information to aid in assignment, a 3D amide-amide SOFAST NOESY-HMQC experiment (pulse sequence sfnoesyhmqc3d.HnHa-NHn) was collected with 0.2 s recycle delay, 300 msec mixing time, ^15^N indirect acquisition time of 20.5 msec, and ^1^H indirect acquisition time of 16.3 msec (Rossi et al. 2016). Nuclear Overhauser effect (NOE) cross-peaks were assigned manually. All data were processed in NMRPipe and analyzed in NMRFAM-SPARKY on NMRBox (Yamazaki et al. 1994; Delaglio et al. 1995; Lee et al. 2015; Maciejewski et al. 2017). Assigned chemical shifts were input into TALOS-N to calculate secondary structure index (SSI) and random coil index order parameters (RCI-S^2^) (Shen and Bax 2013).

### Biophysical characterization of *in vitro* refolded ZAG

To verify that human ZAG, refolded *in vitro* from *E. coli* inclusion bodies, adopted its native conformation, we characterized its oligomerization state, secondary structure characteristics, and thermal stability prior to solution NMR experiments. Consistent with previous reports (Kumar et al. 2013), size-exclusion chromatography revealed that *in vitro* refolded ZAG eluted as a single, monodisperse peak with a molecular weight consistent with a monomeric species (Supplementary Fig. 1A). Protein purity was verified by SDS-PAGE, which revealed a single, well-defined band near the expected molecular weight of ~​32 kDa (Supplementary Fig. 1B). The far-UV CD spectrum of refolded ZAG exhibited a distinct minimum at 218 nm, characteristic of a predominantly β-sheet structure, alongside minor minima at 208 nm and 222 nm corresponding to its α-helical elements (Supplementary Fig. 1C). The CD-derived secondary structure composition is consistent with the predicted MHC-I-like fold (Gorga et al. 1989). NanoDSF of refolded ZAG revealed a melting temperature of 63.1 °C (Supplementary Fig. 1D), consistent with the previously reported thermal stability of *in vitro* refolded ZAG and endogenous ZAG purified from breast cyst fluid (Sánchez et al. 1997b; Kennedy et al. 2001). Together, these biophysical results demonstrate that the *in vitro* refolding protocol yields pure, monomeric, and correctly folded U-[^2^H,^15^N,^13^C]-labeled human ZAG, establishing the sample’s suitability for solution-state NMR spectroscopy.

## Extent of assignments and data deposition

**Fig. 1:**
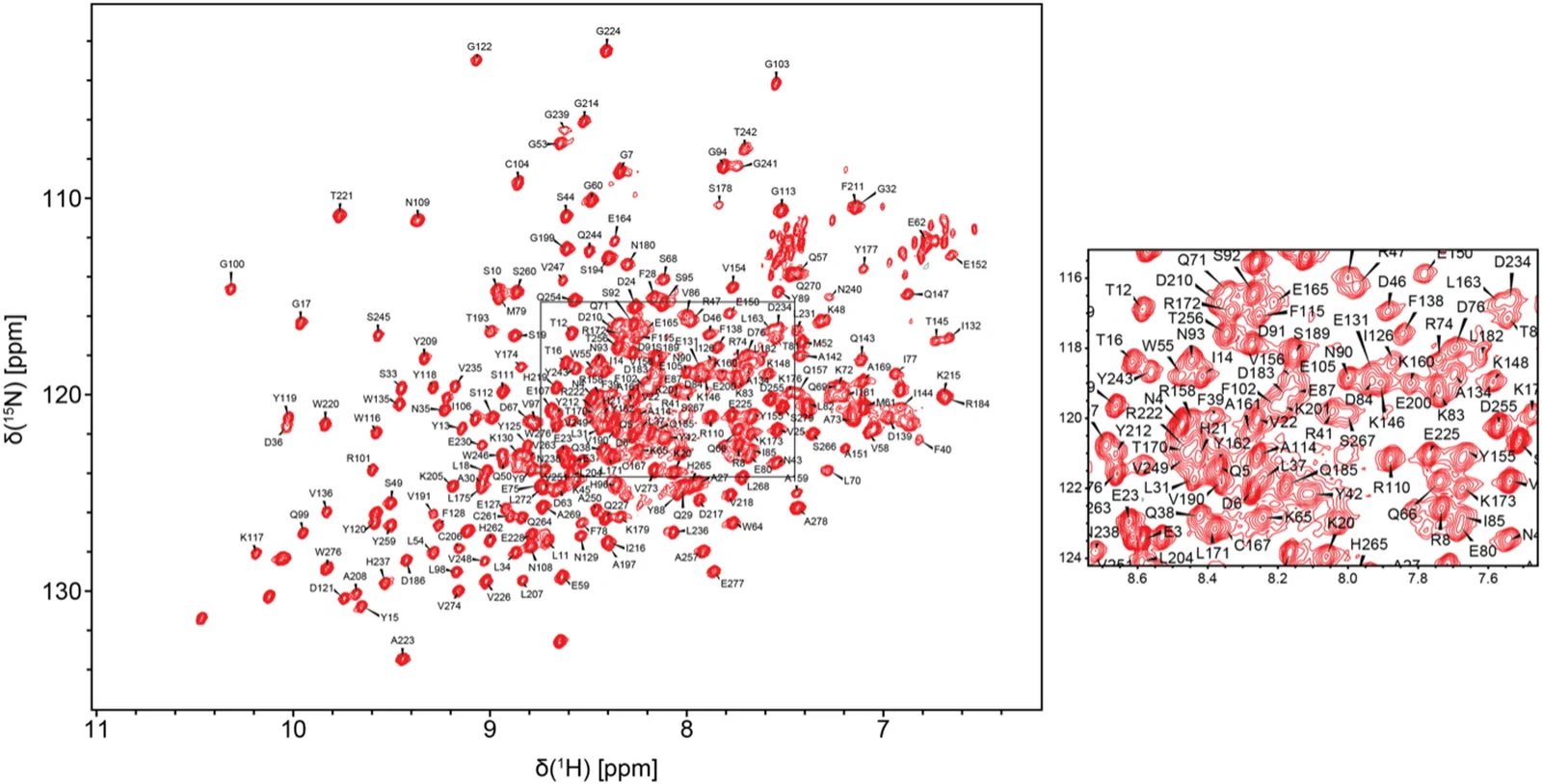
2D ^1^H-^15^ N TROSY-HSQC spectrum of 600 µM U-[^2^H, ^15^N, ^13^C]-labeled human ZAG acquired at ^1^H field of 800 MHz at 25 °C and pH 6.5. Each NMR peak is labeled with its assigned amino acid and residue number

The 2D ^1^H-^15^N TROSY-HSQC spectrum of U-[^2^H,^15^N,^13^C]-labeled ZAG exhibits excellent chemical shift dispersion (Fig. 1), characteristic of a well-folded β-sheet-rich protein and in line with NMR spectral quality reported for classical MHC-I molecules (Varani et al. 2007; Yanaka and Sugase 2017; McShan et al. 2018, 2023). Backbone resonances (^1^H_N_, ^15^N, ^13^Cα, ^13^Cβ, and ^13^C’) of ZAG were assigned by standard sequential assignment strategies using TROSY-based 3D HNCA, HNCO, HNCACB, HN(CO)CACB, and amide-amide NOESY experiments (Frueh 2014) (Supplementary Fig. 2). Assigned chemical shifts have been deposited in the Biological Magnetic Resonance Bank (Ulrich et al. 2008) under accession ID 54,025. Excluding the N-terminal Met and 17 prolines, 247 out of a possible 261 NMR peaks were assigned in the 2D ^1^H-^15^​N TROSY-HSQC spectrum of ZAG (Fig. 1), corresponding to ~ 94.6% of ZAG backbone amide groups. Amide resonances for 14 residues (Q2, R56, K123, D124, A141, W149, E166, V192, H195, Q196, K202, K203, R232, G233) could not be assigned due to lack of ^1^H_N_,^15^N, ^13^C_α_, or ^13^C_β_ resonances. The unassigned ZAG residues were primarily localized to disordered loops or terminal regions of secondary structure elements. Assignment of these residues may have been hindered by incomplete back-exchange or by line broadening arising from conformational exchange on the intermediate timescale. Backbone amide-amide nuclear Overhauser effect (NOE) patterns derived from 3D SOFAST NOESY-HMQC experiments (a total of 856 non-diagonal NOE cross-peaks were assigned) are largely in agreement with the ZAG crystal structure (Supplementary Fig. 3) (Sánchez et al. 1999; Rossi et al. 2016).

## Comparison of SSI and RCI-S^2^ values between ZAG and a class I MHC-I molecule

**Fig. 2:**
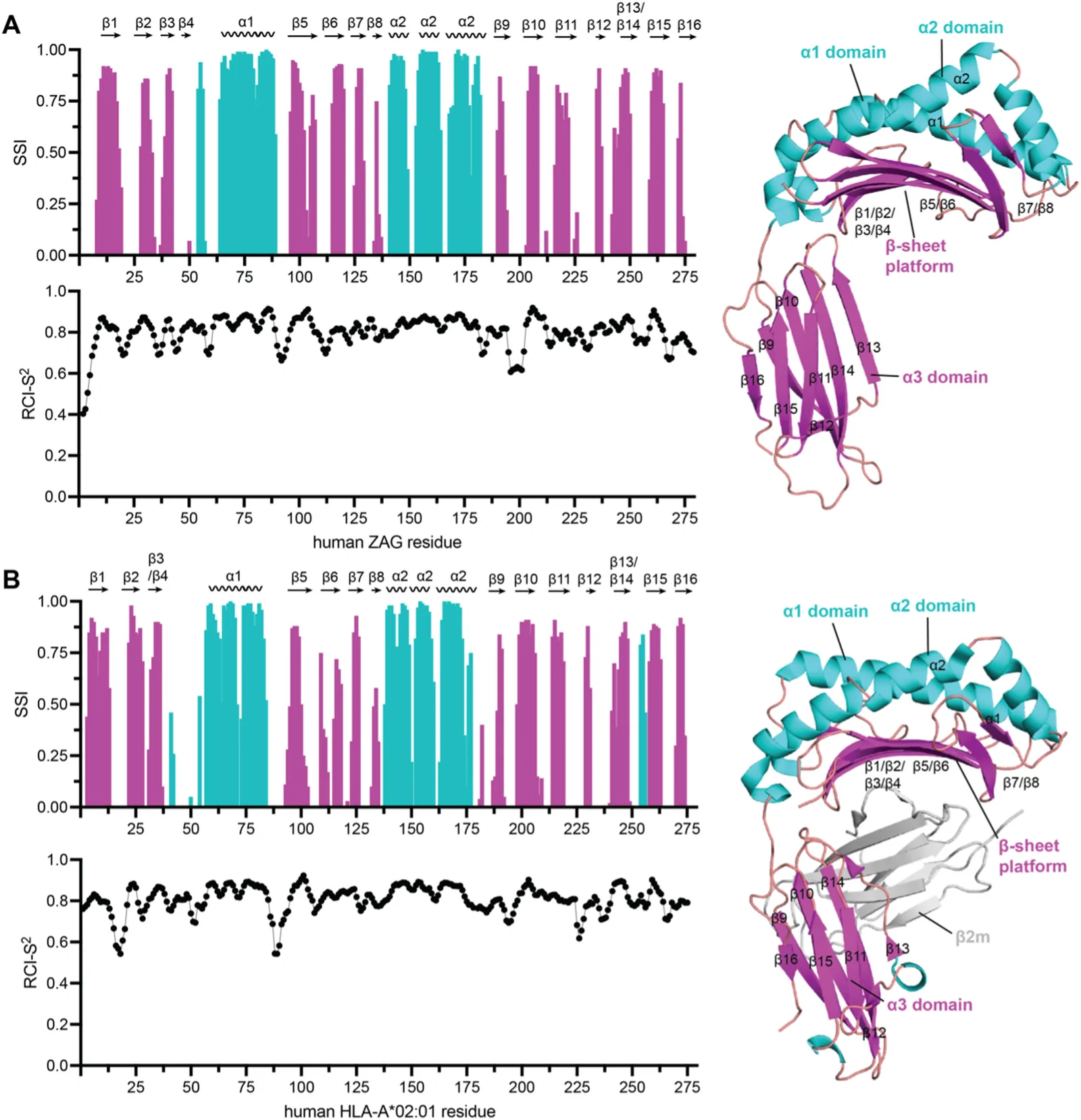
TALOS-N calculated SSI and RCI-S^2^ values derived from assigned ^1^H_N_, ^15^N, ^13^C_α_, ^13^C_β_, and ^13^C’ chemical shifts of (A) human ZAG (described in this work, apo state, doesn’t bind β2-microglobulin), and (B) HLA-A*02:01 (described previously (McShan et al. 2019), TAX9 peptide and β2-microglobulin bound). Crystal structures of ZAG (PDB ID 1ZAG) (Sánchez et al. 1999) and HLA-A*02:01 (PDB ID 1DUZ) (Khan et al. 2000) with bound β2-microglobulin shown in gray) are shown for reference. α-helices and β-strands are colored cyan and purple, respectively. Short breaks in the α2 helix of ZAG and HLA-A*02:01 were identified by TALOS-N from backbone chemical shifts. Although some assignments are missing near these breaks, assigned residues flanking them also show decreasing SSI values, indicating that the breaks are not solely the result of missing data

Given that human ZAG and the class I MHC-I molecule HLA-A*02:01 share 37.7% sequence identity and structural homology with Cα RMSD of 2.56 Å (Supplementary Fig. 4, Fig. 2A, B), we compared solution-state NMR chemical shift-derived characteristics between the two proteins. TALOS-N (Shen and Bax 2015) was used to calculate secondary structure index (SSI) and random coil index order parameter (RCI-S^2^) values from the assigned ^1^H_N_, ^15^N, ^13^Cα, ^13^C_β_, and ^13^C’ chemical shifts of human ZAG (unbound apo state, reported here) and HLA-A*02:01 (TAX9 peptide and β2m-bound state, reported in (McShan et al. 2019). ZAG and HLA-A*02:01 exhibit essentially identical secondary structure characteristics in solution with each showing the canonical α1/α2 helices stabilized by the 8-stranded β-sheet platform, plus a separate α3 immunoglobin-like β-sandwich domain (Fig. 2A, B). RCI-S² order parameters are also comparably high and uniform across most residues in both proteins (roughly 0.75–0.85), meaning ZAG is about as rigid and ordered as HLA-A*02:01 in solution, despite ZAG being unbound (no peptide, no β2m) and having diverged functionally to the point of no longer binding peptide antigens or T cell receptors (Fig. 2A, B).

Overall, the backbone resonance assignments reported here establish a foundation for future investigations into ZAG’s structure, conformational dynamics, and biomolecular interactions, including comparisons with classical and non-classical MHC-I molecules, as well as mapping its complexes with metal ions, lipids, proteins, and nucleic acids.

## Supplementary Information

Below is the link to the electronic supplementary material.

Supplementary Material 1

## Data Availability

Backbone chemical shift assignments for human ZAG have been deposited to the Biological Magnetic Resonance Data Bank (BMRB) under accession ID 54025. Backbone chemical shift assignments for HLA-A*02:01 were previously reported under BMRB ID 27631 (McShan et al. 2019).
