# Supplementary material for: ¹H, ¹³C, and ¹⁵N backbone resonance assignments of zinc-α2-glycoprotein, a lipid-mobilizing protein structurally related to class I HLA molecules
Source: Biomol NMR Assign. 2026 Nov 9;20(1):35. doi: 10.1007/s12104-026-10283-4 (PMC13748185; doi:10.1007/s12104-026-10283-4)
Supplement: Supplementary file 1 [file 12104_2026_10283_MOESM1_ESM.docx]

**Supplementary Information**

**¹H, ¹³C, and ¹⁵N backbone resonance assignments of zinc-α2-glycoprotein, a lipid-mobilizing protein structurally related to class I HLA molecules**

Elizabeth A. Corbin^1‡^, Uri Z. Miles^1‡^, Hongwei Wu^1^, Andrew C. McShan^1*^

^1^School of Chemistry and Biochemistry, Georgia Institute of Technology, Atlanta, GA 30332, USA

^‡^These authors contributed equally to this work.

^*^Correspondence: Andrew C. McShan ([andrew.mcshan@chemistry.gatech.edu](mailto:andrew.mcshan@chemistry.gatech.edu))

**Supplementary Fig 1*.*** Biophysical characterization of *in vitro* refolded human ZAG. (A) Size-exclusion chromatography of *in vitro* refolded ZAG performed using a HiLoad 16/600 Superdex 75 pg column with a flow rate of 1 mL/min. A standard curve performed using a gel filtration calibration kit is plotted onto the SEC chromatogram (black dots) for protein molecular weight reference. (B) SDS-PAGE of SEC purified *in vitro* refolded ZAG. The molecular weight ladder used was PageRuler Unstained Protein Ladder (Thermo Scientific #26614). (C) Far-UV circular dichroism spectra of 0.5 mg/mL ZAG in molar ellipticity, θ, with units deg · cm^2^ · dmol^-1^. (D) First derivative of the nanoDSF denaturation spectra of 10 µM *in vitro* refolded ZAG. Fitted melting temperature (T_m_) is provided as mean ± standard deviation for three replicates.

**Supplementary Fig 2*.*** Example of 3D assignments strip plots obtained for 600 μM U-[^2^H, ^15^N, ^13^C]-labeled human ZAG acquired at ^1^H field of 800 MHz at 25°C and pH 6.5. ^13^C_aliphatic_-^1^H_N_ strips from a 3D TROSY-HNCACB experiment showing sequential assignment of residues R172 to Y177 on the α2 domain.

**Supplementary Fig 3*.*** Backbone amide-amide NOE cross-peaks (obtained from 3D amide-amide SOFAST NOESY-HMQC experiment (pulse sequence sfnoesyhmqc3d.HnHa-NHn with 300 msec mixing time) mapped onto the ZAG X-ray structure (PDB ID 1ZAG).

**Supplementary Fig 4*.*** Amino acid sequence alignment of ZAG (UniProt #P25311) and HLA-A*02:01 (UniProt # P04439) performed in Clustal Omega v1.2.4 and analyzed in ESPript v3.0. Red box, white character → Strict identity. Red character → Similarity in amino acid type. Secondary structure elements shown are based on the ZAG crystal structure (PDB ID 1ZAG). Residue numbering corresponds to the ZAG sequence nomenclature used for the resonance assignments.
